# *Clostridioides difficile* infection and antibiotic prescribing at a regional hospital in Australia: a case–control study

**DOI:** 10.1017/ash.2025.10113

**Published:** 2025-09-01

**Authors:** Sarah Elizabeth Alland, Bianca Mills, Michelle Bolte, Colleen Ma, Sally Munnoch, Matthew Kelly

**Affiliations:** 1 National Centre for Epidemiology and Population Health, Australian National University, Canberra, ACT, Australia; 2 Health Protection Service, Hunter New England Local Health District, Newcastle, NSW, Australia; 3 Infection Prevention Service, Hunter New England Local Health District, Newcastle, NSW, Australia; 4 Medical Department, Hunter New England Local Health District, Tamworth, NSW, Australia

## Abstract

**Background:**

*Clostridioides difficile* infections (CDI) increased at a large, regional hospital in New South Wales, Australia, in 2021, coinciding with an increase at hospitals Australia wide. We aimed to investigate the association between antibiotic prescribing practices and hospital-acquired CDI at the hospital to inform antimicrobial stewardship (AMS) programs.

**Methods:**

We conducted a retrospective case–control study for the period July 1, 2018, and June 30, 2022. Seventy hospital-acquired CDI cases were selected for the study. Cases were matched on a 1:2 basis with randomly selected controls based on date of hospitalization and age group. We conducted a multivariable analysis to explore possible risk factors for infection. We compared cases and controls who were administered antibiotics to determine if rates of inappropriate antibiotic prescriptions differed between cases and controls.

**Results:**

The multivariable model found that use of cephalosporins (third, fourth, or fifth generation) (adjusted odds ratio (aOR) 3.82, 95% confidence interval (CI) 1.35–10.84), use of penicillins (broad or extended broad spectrum) (aOR 5.79, 95% CI 2.15–15.58), and increased complexity of comorbidities (aOR 1.22, 95% CI 1.02–1.45) were independently associated with CDI. In patients who had antibiotics administered during their admission, inappropriate antibiotic prescribing (OR 5.68, 95% CI 1.95–16.48) and non-compliance with antibiotic prescribing guidelines (OR 5.01, 95% CI 1.07–14.76) were associated with CDI.

**Conclusions:**

Our study showed that antibiotic prescribing practices were associated with hospital-acquired CDI at the hospital during the study period. The results reinforce the importance of compliance with antibiotic guidelines and provide further evidence for AMS programs to reduce CDI.

## Introduction

*Clostridioides difficile*, is an anaerobic, gram-positive, spore-forming bacillus that can cause gastrointestinal disease and is a major cause of nosocomial infections.^1^ Hospital-acquired *C. difficile* infection (CDI) is associated with negative outcomes for patients and the health system. CDI can lead to reduced quality of life for patients, associated with ongoing diarrhea, recurrent infections and loss of dignity.^2^ Recent studies have shown that the devasting impacts on patients’ overall quality of life continues well beyond infection clearance as patients fear the infection will return.^3^ Estimates suggest that CDI is associated with infection-related mortality of 5% and all-cause mortality of 15%–20% within a month of diagnosis.^1,2^ Increased financial costs to the health system and impacts on patient flow also result from patients requiring additional treatment and stays in hospital nearly five times longer than the average length of stay.^4,5^

There is strong evidence that antibiotic use is associated with CDI.^1,2,6–8^ All antibiotic classes can be associated with CDI, but association has commonly been found with use of broad-spectrum penicillins, cephalosporins, and fluroquinolones.^1,2^ There is also evidence of increased CDI risk associated with the number, dose, and duration of antibiotics.^2^ Antimicrobial stewardship (AMS) and compliance with antimicrobial prescribing guidelines are key strategies for minimizing hospital-acquired CDI.^5^ However, there is little evidence on the association between AMS strategies, including appropriate use of antibiotics and compliance with antibiotic guidelines, and reductions in CDI.

Routine surveillance at a hospital in regional New South Wales (NSW), Australia identified a large increase in healthcare-associated (HCA), healthcare facility (HCF)-onset CDI^9^ in 2021. Although an increase was observed in CDI data Australia wide,^5^ the observed increase at the hospital prompted consideration of factors that may be contributing to CDI rates at the hospital.^10^ There was a suspicion that inappropriate antibiotic use^11^ may be contributing to CDI at the hospital. The aim of this study was to identify areas for intervention to reduce CDI rates at the hospital and build the evidence base for AMS programs by 1) investigating risk factors associated with HCA, HCF-onset CDI and 2) for patients who were prescribed antibiotics, assessing the association between AMS targeted antibiotic prescribing practices and CDI.

## Methods

### Study design and setting

A case–control study was conducted at the hospital to retrospectively investigate HCA HCF-onset CDI in patients between July 1, 2018, and June 30, 2022. The hospital is a 380-bed public rural referral hospital administered by Hunter New England Local Health District (HNELHD).

### Sample size

Sample size was determined based on antibiotic usage as the main exposure of interest, with a review of previous literature estimating that antibiotic usage was expected to increase the odds of CDI by approximately 3.9 times.^12–17^ Sample size calculation estimated that at least 50 patients were required per case and control group to achieve a confidence level of 95% and power of 90%.^18^

### Study population

Inpatients at the study hospital during the study period who had an infection that met the Australian Commission on Safety and Quality in Health Care (ACSQHC) definition of a healthcare-associated healthcare facility-onset CDI^9^ during that admission were included as cases in the analysis. HNELHD uses a laboratory system for identification of CDI cases.^9^ Eighty-one patients with dates of admission to the hospital in the study period were recorded in HNELHD systems as having HCA HCF-onset CDI. After reviewing patient medical records, 11 patients were identified as having CDI symptoms within 48 hours of admission or having had a CDI diagnosis within eight weeks prior to admission and were excluded. The remaining 70 patients were included in the analysis.

Cases were pairwise matched to randomly selected controls on a 1:2 basis. Controls were matched based on date of hospitalization (± 14 d) and age group (30–49 yr, 50–69 yr, and 70+ years).

Controls were selected from inpatients at the hospital during the study period who were admitted for more than 48 hours and did not have a positive *C. difficile* test while admitted. Controls were excluded if they were admitted to the mental health ward, they had diarrheal symptoms recorded during the admission, or they had a record of a having CDI within eight weeks prior to admission.

### Data extraction

Cases were identified from routinely collected HNELHD CDI surveillance data recorded in ICNet (Baxter International, USA). Control data were extracted from i.PM (Indizium, Singapore), HNELHD’s patient information system. Sex, admission and discharge dates, proton pump inhibitor (PPI) use, admitting medical officer, antibiotic prescription details, and prior hospital admission/s in the previous eight weeks were extracted from medical records for the patient admission. Data extracted from medical records were recorded in the REDCap (Research Electronic Data Capture) tools^20^ hosted at HNELHD. Comorbidity data was extracted from ICD-10AM diagnosis codes assigned to the admission and all HNELHD admissions for the patient in the previous five years as recorded in admitted patient data stored in the NSW Health Enterprise Data Warehouse (EDWARD).

### Definitions

Length of hospital exposure was calculated as the number of days from the start of the case of care until the time of the first positive *C. difficile* test for cases, or until the time of discharge from the case of care for controls. Length of stay was calculated as the number of days from the start of the case of care until the time of discharge from the case of care. Mortality was defined as death within eight weeks of hospitalization, regardless of cause of death. Patients were coded as either surgical patients or nonsurgical patients based on the specialty of the admitting medical officer for the admission.

Antibiotics and PPI were classified as being used if the patient had any antibacterial agent or PPI administered, respectively, during the admission at the hospital and before the time of *C. difficile* diagnosis for cases. The number of antibiotics used was the number of antibacterial agents that the study patient was administered during this time. Antibiotic classes were defined as being used if the patient had any antibacterial agent in that class administered during the admission, as defined by the World Health Organization’s anatomical therapeutic chemical classification system.^21^

Appropriateness of antibiotic prescribing and compliance with prescribing guidelines were included as antibiotic practices targeted by AMS programs. The practices were assessed for all antibiotics administered during the admission using the Hospital National Antimicrobial Prescribing Survey (NAPS) definition matrices.^11^ Compliance with guidelines is considered when assessing for appropriateness of antibiotic prescribing; however, an antibiotic can be considered appropriate but non-compliant with guidelines and vice versa.^11^ Study participants were assigned a comorbidity score, representing the number and complexity of their comorbidities, using an ICD-10AM adaption of the Charlson Comorbidity Index (CCI).^23^

### Data analysis

Data analysis was conducted using R version 4.2.3 and Stata/IC 14.2. Date of admission of cases and age group of cases and controls were described. Patient outcomes were described, with univariable conditional logistic regression conducted to assess the difference in length of stay and mortality between cases and controls.

Potential CDI risk factors were described for cases and controls, with categorical variables described by frequency and percentage, and continuous variables described by their median and interquartile range for each group. Univariable conditional logistic regression were conducted to assess the association of potential risk factors with CDI. Multivariable analysis was performed using a backward stepwise approach and all variables were included except fluoroquinolone and lincosamide use, which were excluded due to low prescription numbers. Variables with a *p* value less than .05 in the final model were considered statistically significant.

Cases who were administered antibiotics and had at least one matched control who was administered antibiotics were included in a subset of the study population. Univariable conditional logistic regression was performed on this subset to assess the association between inappropriate antibiotic prescribing and non-compliance with antibiotic prescribing guidelines and CDI.

### Ethics

This project was assessed as negligible risk and authorized as not requiring approval by the Hunter New England Human Research Ethics Committee (AU 2021 03-18).

## Results

### Study population

**Figure 1.**
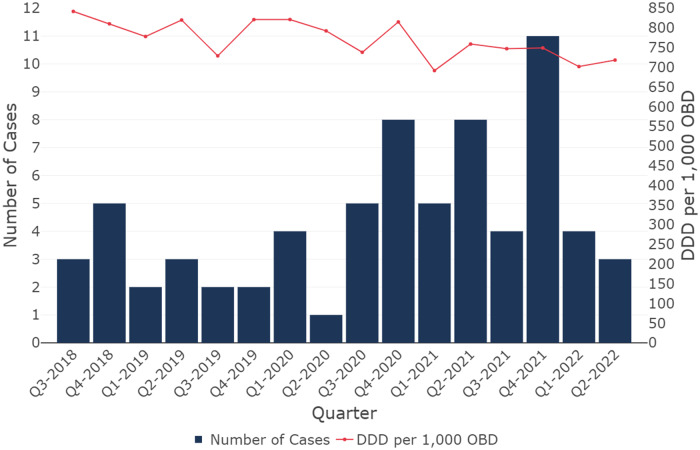
Number of CDI cases diagnosed and rate of Defined Daily Doses (DDD) of antibacterials per 1,000 Occupied Bed Days (OBD)^a^ at the study hospital, by quarter, 1 July 2018 to 30 June 2022. Source: National Antimicrobial Utilisation Program^10^.

Seventy cases and 140 controls were recruited. Most cases (68.6%) were diagnosed with CDI in the latter half of the study period, reflecting the increase in CDI diagnoses observed at the study hospital at this time (figure 1). The median number of diagnoses was four per quarter, with a peak of eleven diagnoses in Quarter 4 2021. Antibacterial use at the hospital remained relatively stable over the study period, with average quarterly use of 770.86 defined daily doses (DDD)^21^ per 1,000 occupied bed days (OBD).

Most cases (72.9%) were in the 70+ age group (table 1). The median age of cases was 76 years (interquartile range 68.2–84.8 yr), and the median age of controls was 75 years (interquartile range 69–84 yr). Cases had a longer median inpatient length of stay at the hospital at 23.03 days compared to controls at 7.05 days (*p* <.001). Cases had mortality of 18.6% and controls had mortality of 12.1%, but the difference was not statistically significant (*p* = .22).

**Table 1.** Age distribution and outcomes of cases and controls

Variable		Cases *n = 70*	Controls *n = 140*
**Age group *n (%)***			
	30–49 years	3 (4.3%)	6 (4.3%)
	50–69 years	16 (22.9%)	31 (22.1%)
	70+ years	51 (72.9%)	103 (73.6%)
**Outcomes**			
	Length of stay (days) Median (Interquartile Range (IQR))^*^	23.03 (12.60–35.30)	7.05 (4.39–13.80)
	Death <8 weeks after hospitalisation n (%)^*^	13 (18.6%)	17 (12.1%)

* There was a statistically significant difference in length of stay (*p* <.001) but no statistically significant difference in death less than 8 week after hospitalisation (*p* = .22) between cases and controls.

### Risk factor analysis

In the univariable analyses, prior hospital admission, antibiotic use, number of antibiotics prescribed, use of cephalosporins (first or second generation), use of cephalosporins (third, fourth or fifth generation), use of penicillins (broad or extended broad spectrum), and CCI score were significantly associated with CDI (Table 2). Adjusting for the interaction with other risk factors, the multivariable analysis found use of cephalosporins (third, fourth or fifth generation (adjusted odds ratio (aOR) 3.82, 95% CI 1.35–10.84), use of penicillins (broad or extended broad spectrum) (aOR 5.79, 95% CI 2.15–15.58), and a higher CCI score (aOR 1.22, 95% CI 1.02–1.45) to be independently associated with CDI (Table 3).

**Table 2.** Univariable analysis of risk factors for *C. difficile* infection, by case status

Variable	Cases *n = 70*	Controls *n = 140*	Univariable analysis	
			Odds ratio (95% CI)	*p* value
Female *n (%)*	39 (55.7%)	74 (52.9%)	1.11 (0.64–1.91)	0.713
Length of hospital exposure (days) *Median (IQR)*	9.85 (5.75–15.80)	7.05 (4.39–13.80)	1.00 (0.98–1.01)	0.550
Prior admission<8 weeks *n (%)*	31 (44.3%)	43 (30.7%)	1.94 (1.02–3.68)	0.043
Antibiotic use *n (%)*	65 (92.9%)	77 (55.0%)	13.89 (4.24–45.55)	<0.001
Number of antibiotics *Median (IQR)*	3 (2–4)	1 (0–2)	1.61 (1.33–1.95)	<0.001
Cephalosporins—first or second generation *n (%)*	23 (32.9%)	28 (20.0%)	1.94 (1.02–3.69)	0.045
Cephalosporins—third, fourth or fifth generation *n (%)*	29 (41.4%)	15 (10.7%)	6.04 (2.73–13.35)	<0.001
Penicillins—broad or extended broad spectrum *n (%)*	36 (51.4%)	20 (14.3%)	5.82 (2.86–11.86)	<0.001
Fluoroquinolones *n (%)*	5 (7.1%)	3 (2.1%)	4.33 (.82–22.82)	0.084
Lincosamides *n (%)*	4 (5.7%)	4 (2.9%)	2.00 (.50–8.00)	0.327
Surgical patient *n (%)*	33 (47.1%)	55 (39.3%)	1.38 (.77–2.47)	0.278
Proton pump inhibitor (PPI) use *n (%)*	41 (58.6%)	77 (55.0%)	1.14 (.66–1.98)	0.640
Charlson Comorbidity Index (CCI) score *Median (IQR)*	3 (2–5)	2 (1–4)	1.13 (1.01–1.26)	0.035

**Table 3.** Multivariable analysis of risk factors for *C. difficile* infection, by case status

Variable^a^	Adjusted odds ratio (95% CI)	*p* value
Length of hospital exposure (days) *Median (IQR)*	.98 (.94–1.01)	0.194
Prior admission<8 weeks *n (%)*	2.48 (.98–6.23)	0.054
Antibiotic use *n (%)*	3.71 (.88–15.67)	0.074
Cephalosporins—first or second generation *n (%)*	2.08 (.87–4.99)	0.099
Cephalosporins—third, fourth or fifth generation *n (%)*	3.82 (1.35–10.84)	0.012
Penicillins—broad or extended broad spectrum *n (%)*	5.79 (2.15–15.58)	<0.001
Charlson Comorbidity Index (CCI) score *Median (IQR)*	1.22 (1.02–1.45)	0.029

a All variables assessed by univariable analysis (Table 2) were included in the multivariable analysis, except for fluroquinolones and lincosamides which were excluded due to low prescription numbers. Other variables not shown in this table were removed from the multivariable model through backward stepwise regression.

### Antibiotic prescribing practices

Of the 65 cases who had antibiotics administered during admission, 53 cases had at least one matched control who was also administered antibiotics during admission. As such, 53 cases and 74 controls were included in the analysis of antibiotic prescribing practices. Inappropriate antibiotic prescribing (Odds Ratio (OR) 5.68, 95% CI 1.95–16.48) and non-compliance with antibiotic prescribing guidelines (OR 5.01, 95% CI 1.70–14.76) were found to be associated with CDI in patients who were administered antibiotics (Table 4).

**Table 4.** Analysis of the difference in antibiotic prescribing practices between cases and controls who were prescribed antibiotics

	Cases *n = 53*	Controls *n = 74*	Odds ratio (95% CI)	*p* value
Inappropriate antibiotic prescription *n (%)*	43 (86.8%)	40 (54.1%)	5.68 (1.95–16.48)	0.001
Non-compliant antibiotic prescription *n (%)*	44 (83.0%)	41 (55.4%)	5.01 (1.70–14.76)	0.003

## Discussion

CDI is a major cause of nosocomial infections and in many cases is considered preventable.^24^ CDI rates increased in Australian hospitals in late 2021.^5^ Surveillance analysis hypothesizes that COVID-19 activity in hospitals may have put pressure on health systems, leading to higher rates of infections.^5^ However, the reasons for the increase have not been systematically analyzed.

The increase in CDI was observed locally at a large, regional hospital in New South Wales, Australia. Overall antibiotic usage at the hospital was high but relatively stable during this period. This prompted consideration of the ways that antibiotics are prescribed, among other factors, to gather evidence to support AMS at the hospital. AMS has long been a priority in Australian healthcare settings to reduce the harmful impacts of inappropriate antimicrobial use^25^; however, during the study period, the hospital had no formal resourced AMS team and relied on a self-moderated system of appropriate prescribing. An AMS policy, with a standardized formulary existed, but the facility had no routine restriction of antibiotics before or during the study period.

### Patient risk factors

This study found that antibiotic usage was associated with CDI at the hospital during the study period. Use of any antibiotics was found to increase the odds of CDI by approximately 14 times in univariate analysis. However, the multivariable analysis showed that it was the use of specific types of antibiotics that was associated with the outcome of CDI, rather than the use of antibiotics generally. Broad-spectrum antibiotics cause a larger disruption of intestinal microflora than narrow-spectrum antibiotics and have been commonly associated with CDI in other studies.^6^ The odds of CDI were approximately 4 times higher for patients who were prescribed a third, fourth or fifth generation cephalosporin and 6 times higher for patients who were prescribed a broad or extended broad spectrum penicillin. In comparison, a previous meta-analysis found a similar association between cephalosporins and hospital-acquired CDI, with the estimated odds ratio ranging from 2.14 for fourth generation cephalosporins to 3.20 for third generation cephalosporins.^19^ The meta-analysis also found an association between penicillin combinations and hospital-acquired CDI with an estimated odds ratio of 1.45, slightly lower than estimated in our analysis.^19^ Use of fluoroquinolones and lincosamides were not included in the multivariable analysis because use of these antibiotics was low at the hospital. Univariable analysis suggested that use of these antibiotics may increase the odds of CDI but the effects were not statistically significant. The association of fluoroquinolone use with CDI is supported by other studies.^1,2,12,19,26,27^ Further research is required with larger study populations to better understand the effect of lincosamides on CDI.

National Antimicrobial Utilisation Surveillance Program (NAUSP) data showed that the average usage of broad and extended broad spectrum penicillins at the hospital across the study period was above the target rate of 30 DDD per 1 000 OBD established as part of HNELHD’s AMS program.^10^ There was particularly high usage of amoxicillin + clavulanic acid (monthly average of 87.7 DDD per 1 000 OBD), and piperacillin + tazobactam (monthly average of 39.4 DDD per 1 000 OBD).^10^ The average usage of cephalosporins was below the target rate but usage of cefazolin, ceftriaxone and cefalexin was well above the target rate (monthly averages of 93.0, 46.1 and 47.7 DDD per 1 000 OBD, respectively).^10^ Interventions to reduce the usage of these antibiotics at the hospital could help to reduce CDI acquisition at the hospital.

Appropriate usage of antibiotics is a key objective of Australia’s National Antimicrobial Resistance Strategy.^28^ Antibiotics are an important tool for the treatment of bacterial infections, including CDI, but inappropriate use of antibiotics leads to amplification of antibiotic resistance and patient harm.^28^ The subanalysis of cases and controls who were administered antibiotics during admission found high rates of inappropriate antibiotic prescribing and non-compliance with antibiotic prescribing guidelines in both cases and controls. However, the rates were considerably higher in cases than controls. Inappropriate prescription increased the odds of CDI by six times and non-compliance with antibiotic guidelines increased the odds of CDI by five times in patients who were administered antibiotics. All reasons for a prescription to be inappropriate or non-compliant were observed for cases and controls, and most prescriptions had multiple reasons for being considered inappropriate or non-compliant. Strategies to promote optimal antibiotic prescribing practices could reduce CDI at the hospital and improve patient outcomes.

### Patient outcomes

Poorer patient outcomes were observed for cases compared to controls, demonstrating the impact of CDI on patients and the hospital, and underscoring the importance of minimizing CDI in hospital settings.

The median length of stay for cases was longer than the reported average length of stay for patients with a *C. difficile* diagnosis in Australian public hospitals (23.03 d compared to 16.58 d).^5^ The difference in length of stay between cases and controls of 15.98 days was toward the upper end of estimates found in international literature.^2^

The all-cause mortality rate for cases at eight weeks postadmission was 18.6% and was similar to estimates in previous studies.^1,2^ The all-cause mortality rate for cases was slightly higher than for controls but the difference was not statistically significant. It is unknown what proportion of this difference can be attributed to CDI and is likely impacted by confounding factors, such as comorbidities.

## Limitations

There were several limitations to the study. The findings of the analysis are representative of patients at the hospital during the study period and may be indicative of risk factors for CDI in similar large, regional hospital settings. However, they may not be representative of CDI in other settings, such as residential care institutions.

All data were obtained from HNELHD record systems. Antibiotic exposure may be underestimated if patients had been prescribed antibiotics in the community prior to their hospital admission, and this was not noted in their hospital record. It is also possible that patients could have had a community-associated CDI prior to their hospital admission that was not recorded in their hospital record. Patients were not screened for colonization of *C. difficile* prior to hospitalization, and no postdischarge surveillance was undertaken to identify patients who may have developed CDI after discharge.

Antibiotic use was grouped by antibiotic class due to the small sample size available for the study. Even so, the use of some antibiotic classes was low, leading to large confidence intervals and low precision for effect estimates.

One case in the 50–69-year age group needed to be matched with a control in the 70+ year age group due to the low number of patients available as controls in the 50–69-year age group and admitted within two weeks of the case. It is not expected that this would have considerably affected the results of the analysis.

Comorbidities were assessed based on ICD-10AM codes recorded with hospital admissions. ICD-10AM codes may underestimate comorbidities because the codes are assigned for administrative purposes and are not intended to be a definitive record of patient comorbidities. Classification of patients as surgical or nonsurgical was based on the specialty of the admitting medical officer. Patient type can vary during admission, and it is possible that some nonsurgical patients could have had a surgical procedure during their admission for which they were prescribed surgical prophylaxis. Despite these limitations, there is no reason to believe that these factors would differ between cases and controls, minimizing concerns of selection bias.

## Conclusion

The study found that the use of specific classes of broad-spectrum antibiotics increases the risk of healthcare-associated healthcare facility onset CDI in hospital inpatients and inappropriate use of these antibiotics was associated with CDI in patients who were administered antibiotics. Inappropriate antibiotic prescriptions and non-compliance with antibiotic guidelines were high at the hospital. Hospital costs and patient morbidity associated with CDI could be reduced with improved adherence to and embedding of AMS practices within the hospital.
